# Acute Toxicity and Ecotoxicological Risk Assessment of Three Volatile Pesticide Additives on the Earthworm—*Eisenia fetida*

**DOI:** 10.3390/ijerph182111232

**Published:** 2021-10-26

**Authors:** Wenqiang Wang, Jing Zhang, Jingya Wu, Ran Yu, Yimin Zhang, Liwei Sun, Yuexiang Gao

**Affiliations:** 1 School of Energy & Environment, Southeast University, Nanjing 210096, China; 220190511@seu.edu.cn (W.W.); 220190584@seu.edu.cn (J.Z.); 220200551@seu.edu.cn (J.W.); yuran@seu.edu.cn (R.Y.); 2 Taihu Lake Water Environment Engineering Research Center (Wuxi), Southeast University, Wuxi 214000, China; 3 Nanjing Institute of Environmental Sciences, Ministry of Ecology and Environment of the People’s Republic of China, Nanjing 210042, China; zym@nies.org

**Keywords:** pesticide adjuvants, *Eisenia fetida*, acute toxicity, growth inhibition, avoidance behavior

## Abstract

Pesticide adjuvants (PAs) are important components of pesticide. Nonetheless, limited information is available regarding their toxic effects on biota in terrestrial ecosystem. In the present study, the mortality, growth inhibition ratio, and avoidance behavior of *Eisenia fetida* were examined to investigate the ecotoxicological effects of toluene, xylene, and trichloroethylene and further their mixture. The 24 h median lethal concentration (24 h-LC_50_) of the three PAs were 300.23, 1190.45, and 5332.36 mg/kg, and the 48 h-LC_50_ values were 221.62, 962.89, and 4522.41 mg/kg, respectively. The mixture exhibited significant synergistic effect on the *E. fetida*. There was significant growth inhibition on *E. fetida* by the tested PAs. The avoidance threshold values of *E. fetida* for the tested PAs were 1100 mg/kg, 250 mg/kg, 5000 mg/kg, and 25% of the mixture, respectively. The results evaluated the toxic effects of the three PAs and their mixture on *E. fetida*, provided a basis for ecotoxicological risk assessment of PAs in the soil ecosystem.

## 1. Introduction

Pesticide adjuvants (PAs) are the general term for other auxiliary substances used in the production and application of pesticide preparations except for the active ingredients. Generally, PAs are added to improve the bioavailability of pesticide formulations by improving the solubility or the compatibility of active ingredients. Since most active ingredients are difficult to use directly, PAs not only account for a significant proportion of pesticide formulations (1~99%), but also comprise a large and heterogeneous group of substances. There are more than 4000 varieties of PAs internationally [1].

Generally, the ecotoxicological risk assessment of pesticides target the deleterious effects of the active ingredient, excluding the potential toxicity of PAs. PAs have long been unregulated and are mistakenly considered as inert additives, devoid of pesticide activity. However, a number of recent researches have revealed that PAs could also be hazardous to ecosystems and human health and even more toxic than the pesticide active ingredients [2,3]. In addition, PAs can affect the profiles of pesticides transport and transformation in ecosystem or generate joint toxicity with pesticide active ingredients. Furthermore, PAs released into the environment can induce ecotoxicity in nontarget organisms including humans through migration and transformation. According to investigations, there are about 1000 PAs that are at less moderately toxic, and half of them have potential risks of teratogenicity, carcinogenicity, and mutagenicity [4]. Alcohol ethoxylates have been reported to modify the structure and permeability of the cell membrane system by interacting with proteins and membrane phospholipids [5]. The neurotoxicity, hepatotoxicity, and development toxicity of toluene and xylene has been well documented [6,7]. Trichloroethylene, as a developmental neurotoxicant, can not only disturb the cell viability and induce apoptosis and necrosis, but also affect the neural proliferation, neurite outgrowth, and differentiation [8]. Additionally, the previous study has documented the toxic effects of certain glyphosate-based formulations (Roundup FG and Mon 8750) on earthworms through lethal toxicity, lysosomal destabilization, and DNA damage [9]. Silwet L-77 has been reported about its significant influence on the survival, colony size, and hatchability of honeybees [10]. Moreover, the organic solvents in PAs take a great proportion, in which toluene, xylene, and trichloroethylene are the most important types [11]. These three PAs are volatile and widely used in agriculture. Additionally, the leakage incidents during production, storage, and transportation have further increased the emission of the three volatile PAs into the ecosystem. At present, the research on the three volatile PAs mainly focuses on their environmental behavior and ecotoxicity in the aquatic environment [12,13]. There are few studies on the ecotoxicity and toxic mechanism of the three volatile PAs to the terrestrial ecosystem, which has an adverse effect on the remediation of sites contaminated by the three volatile PAs.

Earthworms, one of the most prevalent soil macroinvertebrates, inhabit in the soil ecosystem, accounting for 60~80% of the total soil biomass, and play an important role in preserving the productivity of the soil ecosystem by regulating the turnover of mineral nutrients and organic matter, improving soil properties, and maintaining soil structure [14,15]. Earthworms are the base of many food chains and are easily exposed to soil contaminants via their intestine or skin, which can further affect the higher trophic level terrestrial organism populations through food chain. Moreover, they are easy to handle under laboratory conditions and are used to quantify different physiological and behavior parameters. Thus, earthworms are an ideal model bioindicator organism in soil ecotoxicological risk assessment to provide the safety thresholds for various environmental pollutants [16]. *Eisenia fetida* was selected as the model test organism due to its sensitivity to contaminants. The individual levels endpoints, such as mortality, growth inhibition ratio, avoidance behavior, and reproductive rate have been widely used in toxicology tests to investigate the adverse effects of contaminants in the soil ecosystem [17]. There have been standard methods for the lethal test described in the OECD and GB/T 31270.15-2014 guidelines [18,19].

In the present study, the toxicities of three volatile PAs (toluene, xylene, and trichloroethylene) and their ternary mixture on *E. fetida* were assessed by the acute lethal toxicity test, the growth inhibition test, and the avoidance test. The results evaluated the toxic effects of the three volatile PAs and their ternary mixture on *E. fetida* at an individual level and obtained a more comprehensive potential ecotoxicological risk evaluation of PAs on the soil ecosystem.

## 2. Materials and Methods

### 2.1. Chemicals and Reagents

The chemical information of three volatile PAs in the present study is listed in Table 1. Toluene was supplied by Sinopharm Chemical Reagent Co., Ltd. (Shanghai, China) Xylene, trichloroethylene and dimethyl sulfoxide (CAS No. 67-68-5, 99% purity) were obtained from Macklin. Dimethyl sulfoxide was used as a solvent.

### 2.2. Tested Biota

*E. fetida* were obtained from Jiangsu Run long Ecological Agriculture Co., Ltd. They were cultured at room temperature (20 ± 1 °C) in darkness and fed regularly by the cow excrements according to OECD guidelines [18]. A normal mature earthworm with well-developed clitellum, individual wet weight of 300~600 mg, was chosen for tests. *E. fetida* were selected 24 h prior to tests, rinsed with ultra-pure water, and kept on a damp filter paper at 20 ± 1 °C in the dark to eliminate gut contents.

### 2.3. Acute Lethal Toxicity and Growth Inhibition Test

The methods for the lethal test described in the OECD and GB/T 31270.15-2014 guidelines employed an open interface [18,19]. From our pre-test results, they were not suitable for volatile compounds. Therefore, the acute lethal toxicity test and growth inhibition test were performed according to the closed soil microcosm for 48 h [20].

The test device was a flat-bottomed glass tube (ID 26 mm, length 80 mm), which was closed tightly with the aluminum foil to prevent volatilization of PAs. The test matrix was the artificial soil, consisting of 70% quartz sand, 20% kaolin clay, and 10% sphagnum peat moss [18]. The pH was 6.0 ± 0.5 adjusted with calcium carbonate. Ten g of dry soil was placed into the test device and aqueous solution with different concentrations of PAs were evenly spiked, respectively. Furthermore, the ultra-pure water was added to adjust the water-soil weight ratio to 1:4. The test device was closed for 24 h to balance and then the soil was mixed thoroughly with a spoon. One mature earthworm was added to each test device and the test was done with 10 replicates. All test devices containing earthworms were put in a climate room at 20 ± 1 °C under 80 ± 5% relative humidity in the darkness throughout the 48-h incubation period.

The mortality and abnormality were recorded at 24 h and 48 h. The earthworms were regarded dead when no response to gentle mechanical stimulation of the anterior region was observed. Meanwhile, the biomass of each tested earthworm was recorded after 12, 24, 36, and 48 h to evaluate the growth inhibition effects. The PAs concentrations were set up based on results from range-finding test. The concentrations of toluene were prepared as 800, 900, 1000, 1100, and 1200 mg/kg, the concentrations of xylene were prepared as 200, 250, 300, 350, and 400 mg/kg, and the concentrations of trichloroethylene were prepared as 4000, 5000, 6000, 7000, and 8000 mg/kg.

The mixing ratio of three PAs was prepared as 1:1:1 based on the 48 h-LC_50_ values of each chemical derived from separated tests, which were set as 100% mixture stock solution. Then, a series of diluted concentrations of the mixtures were prepared, i.e., 15, 20, 25, 30, and 35%. The experiments were conducted by the same method as in the separated test. The additive index (AI) was calculated to evaluate the mixture toxicity type.

Blank controls (BC) and solvent controls (SC) were prepared with ultra-pure water or dimethyl sulfoxide (except for the trichloroethylene treatment).

### 2.4. Avoidance Test

The six section chamber was employed to assess the avoidance behavior response of earthworms to the PAs in soil [21]. The circular test device had a central chamber with six cut pie-shaped interconnecting compartments into which the test soil was placed, interconnecting holes were located along the bottom of the compartment walls (three per side) and along the bottom of the central chamber (two per side), so that the earthworms can move freely between the six compartments. Then, 350 g of tested soil and control soil, which were moistened by ultra-pure water to obtain 60% of the maximum water holding capacity, were prepared and placed 50 mm to 60 mm into each compartment. Ten earthworms were added to the central chamber, one piece at a time, and the compartment entered by each individual earthworm was recorded. The test device was wrapped tightly in a sealed bag to prevent volatilization of PAs. The test was done with three replicates.

All test devices were set for 48 h at 20 ± 1 °C and a light/dark cycle of between 16 h/8 h under 80 ± 5% relative humidity. At the end of the test, the dividers were positioned to prevent further movement of the earthworms between compartments. The numbers of earthworm in each compartment were recorded and the total number in each treatment within a test device was determined. Each individual earthworm which was sliced inadvertently by the dividers was to be counted as 1/2 earthworm independent of the length of the remaining body.

The PAs concentrations set in the definitive tests were based on results from range-finding test, which were different from the acute lethal test. The concentration of toluene was prepared as 700, 800, 900, 1000, and 1100 mg/kg, the xylene was 50, 100, 150, 200, and 250 mg/kg, and the trichloroethylene was 1000, 2000, 3000, 4000, and 5000 mg/kg.

The mixing of the three volatile PAs was prepared the same as acute lethal toxicity, and the mixed avoidance test was carried out as the same method as the separated test.

### 2.5. Statistical Analysis

All data were presented as mean ± SD (standard deviation). The SPSS software (version 26.0) was used to perform all statistical analyses. A one-way analysis of variance along with the Least-Significant Difference (LSD) test was used to determine the significant differences between volatile PAs treatments at a significance level of *p* < 0.05. The Median lethal concentration (LC_50_) was calculated by the probit analysis on the SPSS software (version 26.0) [22,23]. The results were expressed as the added concentrations.

The growth inhibition rates of earthworms exposed to PAs from the various exposure period were calculated by the equation as follows:

$$I_{n}=\frac{W_{0}-W_{t}}{W_{0}}\times 100\%,$$

where I_n_ represents the growth inhibition rate, and W_0_ represents the average weight of earthworms at the initial day, and W_t_ represents the average weight of earthworms at t h (t ≤ 48 h).

The selection rate, which is less than 10% in the avoidance test soil at 0 and 48 h, indicating that the presence of chemical substances cannot be excluded, is assessed as the avoidance threshold (AT) values [24].

The additive index (AI) method was employed to evaluate the mixture toxicity type of PAs mixture [25]. The biological toxicity (S) of compounds A, B, and C was calculated by the following equation:

$$S=\frac{A_{m}}{A_{i}}+\frac{B_{m}}{B_{i}}+\frac{C_{m}}{C_{i}},$$

where S is the sum of the biological toxicity, A_m_, B_m_, and C_m_ are the LC_50_ and AT values in mixture for compound A, B and C, and A_i_, B_i_, and C_i_ are the individual LC_50_ and AT values for compounds A, B, and C.

The AI was then determined using the following equation:

$$\begin{array}{c} \mathrm{AI}=1/S-1\;\mathrm{for}\;S\;<1.0,\\ \mathrm{AI}=S\;\left(-1\right)+1\;\mathrm{for}\;S\;\geq \;1.0. \end{array}$$

The AI describe the type of mixture toxicity (additive, synergistic, or antagonistic). An AI value less than zero indicates antagonistic toxicity, greater than zero indicates synergistic toxicity, an AI value with confidence interval overlapping zero indicated additive toxicity.

The assessment factor (AF) method and risk quotient (RQ) method were performed to evaluate the ecotoxicological risk of three PAs. The predicted no effect concentration (PNEC) and RQ were calculated by the equation as follows:

$$\begin{array}{c} \mathrm{PNEC}={\mathrm{LC}}_{50}/\mathrm{AF},\\ \mathrm{RQ}=\mathrm{MEC}/\mathrm{PNEC}, \end{array}$$

where the OECD recommended AF value of 1000 was applied; MEC represents the measured environmental concentration of pollutants. RQ describes the risk level (no, low, medium, and high ecotoxicological risk). A RQ value less than 0.01 indicates no ecotoxicological risk, between 0.01 and 0.1 indicates low ecotoxicological risk, between 0.1 and 1 indicates medium ecotoxicological risk, and greater than 1 indicates high ecotoxicological risk.

## 3. Results and Discussion

### 3.1. Acute Lethal Toxicity Test

The pre-tests were performed according to the OECD guideline for 14 days. The concentrations of toluene were prepared as the lower treatments (1000~10,000 mg/kg), there were no mortalities found for worms during the 14 days. However, when the concentrations of toluene were prepared as the higher treatments (30,000~40,000 mg/kg), the symptoms of earthworm poisoning were obvious, and all died within one to two days. After the soils were prepared with a xylene concentration of 30,000 mg/kg for 2 days, 10 earthworms were placed into the soil, and there were no mortalities found for the worms. These results indicated that the open experimental system was not suitable for the volatile compounds. Moreover, there is no emphasis in the OECD and GB/T 31270.15-2014 guidelines for measurement of the chemical concentration, although a few studies pointed out the test concentrations in soil or within the exposed organisms [24,25]. In the present study, due to the short test period, the sealing of the test device, and the limited space, the closed soil microcosm could effectively reduce the loss of PAs, so as to ensure that the earthworms were exposed at the added concentrations, which could be affected at approximate real environmental concentrations. Therefore, the following results were expressed as the added concentrations.

The acute lethal toxicity results of *E. fetida* exposed to each PAs at 24 h and 48 h were presented in Table 2. There were no mortalities found for *E. fetida* both in the blank and solvent control in the tests. The lethal rates showed a clear concentration-mortality relationship for all PAs. Additionally, the three volatile PAs concentrations exhibited different toxicity levels to *E. fetida*.

At 24 h, the xylene showed the highest toxicity to *E. fetida* with an LC_50_ value of 300.23 mg/kg, followed by toluene with 1190.45 mg/kg, and trichloroethylene showed the lowed toxicity with 5332.36 mg/kg, respectively. The decreasing order of toxicity of these three volatile PAs was: xylene > toluene > trichloroethylene. At 48 h, the xylene treatment still exhibited the highest toxicity to *E. fetida* with an LC_50_ value of 221.62 mg/kg, followed by toluene with 962.89 mg/kg and trichloroethylene with 4522.41 mg/kg, respectively. The decreasing order of toxicity of these three volatile PAs was the same as that at 24 h.

Generally, the organic pollutants could be classified into four classes according to the chemical structures: inert chemicals, less inert chemicals, reactive chemicals, and specifically acting chemicals [26]. In the acute toxicity, the inert chemicals and less inert chemicals were called narcosis and the potency of narcosis chemicals depended on their hydrophobicity [27]. The narcosis chemicals were easily adsorbed by biological membranes, and the greater the hydrophobicity was, the easier the narcosis chemical would pass through the biological membranes non-selectively. Then, they would react with biologically active sites through various processes, causing toxicity to organisms. Therefore, the toxicity of narcosis chemicals could be determined by the Octanol-water partition coefficient in the absence of specific mechanisms of toxicity. Three tested volatile PAs are all narcosis chemicals, and the Octanol-water partition coefficient of toluene, xylene, and trichloroethylene are 2.69, 3.12~3.20, and 2.42, respectively. The decreasing order of hydrophobicity of these three volatile PAs was as follows: xylene > toluene > trichloroethylene. This result was consistent with the acute toxicity of these three volatile PAs in the present study.

The mixed toxicity result of the mixture of three PAs are shown in Table 3. The 24 h-LC_50_ of the ternary mixture was 28.10%, in which the concentration of the three PAs was 270.52, 62.26, and 1270.57 mg/kg, respectively. In addition, the 48 h-LC_50_ of the ternary mixture was 20.71%, in which the concentration of the three volatile PAs was 199.41, 45.90, and 936.60 mg/kg, respectively. The AI was 0.49 at 24 h and 0.61 at 48 h, respectively, which revealed that the ternary mixture both displayed a synergistic effect, which exhibited a greater-than-expected impact on the soil ecosystem.

The mixing of components could greatly affect the toxicity of each compound, leading to considerably varied toxic degrees [28]. It is said that the organisms could be subjected to greater chemical synergy in the soil ecosystem since they were chronically exposed to a complex mixture of toxic substances, and the nontarget organisms could be impaired by synergistic interactions, which was unbearable in the inherent surrounding. Mixture components showed synergistic response in that one of the chemicals triggered an alteration in toxicokinetic (i.e., metabolism rates, absorption, and alternative MOA) of the organism [29].

### 3.2. Growth Inhibition Effects

**Figure 1:**
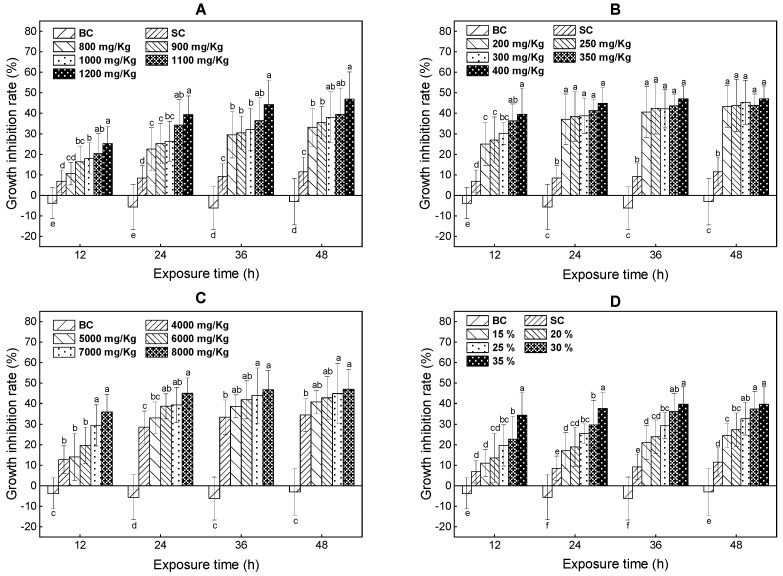
The growth inhibition rate of *E. fetida* exposed to toluene (**A**), xylene (**B**), trichloroethylene (**C**) and ternary mixture (**D**) for 12, 24, 36, and 48 h; BC represents blank controls; SC represents solvent controls; Different lowercase letters indicate significant differences among treatments.

The growth inhibition on *E. fetida* exposed to the tested PAs is presented in Figure 1. The growth inhibition rates of the blank control at 12, 24, 36, and 48 h were negative, indicating that the test condition and the artificial soil were sufficient to sustain earthworm growth during the 48-h exposed period. However, the growth inhibition rate of the solvent control was significantly higher than that of the blank control during the 48-h exposed period (ANOVA, *p* < 0.05), but it was lower than the growth inhibition rates of all tested chemicals. The significant growth inhibition effects were evaluated by the difference between the exposed groups and solvent control for toluene, xylene, and ternary mixture, while for trichloroethylene it was between the exposed groups and the blank control.

For the toluene, at 12 h, the significant growth inhibition rate was first observed at 900 mg/kg (*p* < 0.05). At the 24 to 48 h time points, significant differences were first observed at 800 mg/kg (*p* < 0.05). For the xylene, at 12, 24, 36 and 48 h, the significant growth inhibition rates were first observed at 200 mg/kg (*p* < 0.05). For the trichloroethylene, at 12, 24, 36, and 48 h, the significant growth inhibition rates were first observed at 4000 mg/kg (*p* < 0.05). For the mixture of PAs, at 12 h, the significant growth inhibition rate was first observed at 25% (*p* < 0.05). At other time points, significant differences in the growth inhibition rate from the solvent controls were first observed at 15% (*p* < 0.05).

In general, all tested PAs significantly inhibited the growth of *E. fetida*, and the inhibitory effect significantly increased with the increasing concentrations of tested PAs and exposure time, The decreasing order of growth inhibition of these three volatile PAs was: xylene > toluene > trichloroethylene, which was also consistent with the acute lethal toxicity of these three volatile PAs in the present study. The initial 24 h was the most sensitive period of earthworm biomass changes when the most obvious inhibitory effect occurred.

The results were in accordance with earlier reports on *E. fetida* after being exposed to the flame retardant, neonicotinoid insecticides, and heavy metals [30,31,32]. Additionally, similar biomass adjustments were observed with *E. Andrei* after exposure to Roundup FG and Mon 8750 [9].

As a bioindicator of contamination in soil, the growth inhibition of earthworms could reflect chemical stress, which linked chemical effects to energy of dynamics [33]. In this study, all tested PAs inhibited *E. fetida* growth, and this was possibly correlated with a defensive mechanism by which earthworms reduce food intake to avoid the disorder of membrane systems or the disruption of the cell membrane integrity from contaminants after exposure. Furthermore, it was reported that the detoxification mechanism of earthworms played a significant role in removing exogenous poisonous substances, and that it was an energy metabolism process, which would consume glycogen, lipid, and protein [34]. Moreover, exudation of body fluids was discovered with *E. fetida* after exposure to the tested PAs. Therefore, we suspect that the PAs may influence the normal metabolism of biomacromolecules, trigger the detoxification, and cause body fluids to ooze in *E. fetida* during the 48-h exposed period.

### 3.3. Avoidance Response Effect

**Figure 2:**
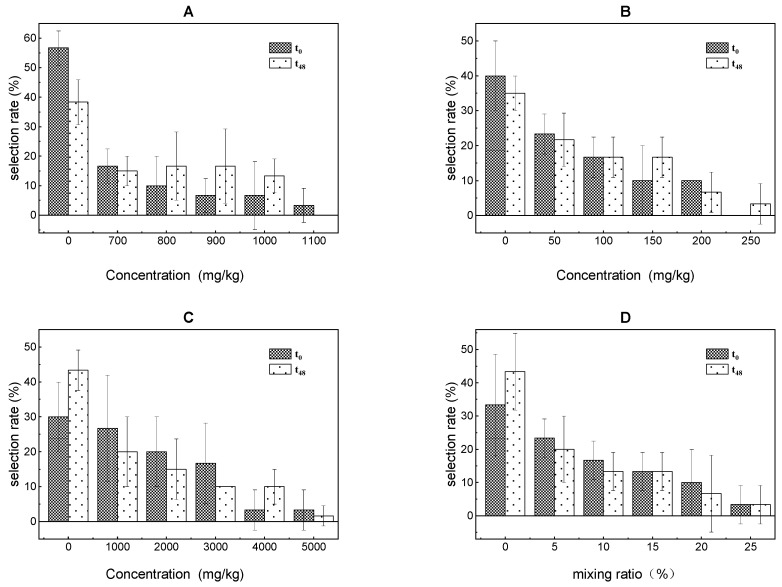
Results of the avoidance test in earthworms after exposure to toluene (**A**), xylene (**B**), trichloroethylene (**C**) and ternary mixture (**D**) for 0 h and 48 h.

The results of the avoidance response test of *E. fetida* to the PAs at 0 h and 48 h were presented in Figure 2. There were no mortalities and disappearances found of *E. fetida* during the test period, therefore the avoidance test results were valid.

It was observed that *E. fetida* returned immediately when they came into compartments with the high treatments and avoided this area at 0 h, showing that *E. fetida* could feel the presence of the PAs and avoided it at the early stage of exposure. At 48 h, the AT values of *E. fetida* for the toluene, xylene, and trichloroethylene treatments were 1100, 250, and 5000 mg/kg, respectively. In addition, the AT value to the mixture of toluene, xylene, and trichloroethylene was 25%, in which the concentration of the three volatile PAs was 240.72, 55.41, and 1130.60 mg/kg, respectively. The AI was 0.50, revealing that the ternary mixture also displayed a synergistic effect in the avoidance test. These results indicated that the habitat or ecological function of volatile PAs contaminated soil had been weakened or changed.

Compared with other endpoints for toxicity assessment, the avoidance test was rapid, inexpensive, and simple to perform, and it has been employed by a number of researchers to evaluate the effects of heavy metals and persistent organic pollutants on *E. fetida* [35,36,37,38]. The results of the present study demonstrated that the behavior of *E. fetida* was affected by the volatile PAs. A similar phenomenon was observed in *E. fetida,* which showed significant abnormal behavior after benomyl, carbendazim, and Lambda-cyhalothrin exposure [39]. The abnormal behavior of *E. fetida* could be based on the strong olfactory senses that make animals identify food and avoid poisonous environments. The results revealed that the odor of the PAs made *E. fetida* avoided them. Similar results were obtained using citrus processing waste, which was attributed to the earthworms’ olfactory capabilities [40]. Therefore, not only direct, but also indirect effects of poisonous substances on *E. fetida* may affect soil ecosystem.

A few studies showed that avoidance response of earthworms could indicate lower AT concentrations than other traditional endpoints, such as mortality, biomass gain/loss, and even reproduction [41,42]. Nonetheless, the results of this study showed that the AT values of *E. fetida* to the toluene, xylene, trichloroethylene, and ternary mixture were higher than the 48 h-LC_50_ value in the acute toxicity test. No significant advantage of avoidance response in sensitivity was observed. Thus, the potential sensitivity of avoidance response of *E. fetidae* was only exhibited to some poisonous substances, not to volatile compounds. This result was consistent with several previous studies [43,44]. During the test period, volatile compounds may interfere with the sensory receptors (earthworms possess chemoreceptors in the prostomium) of earthworms due to their diffusion in all compartments via the gaseous phase. Therefore, for volatile compounds, avoidance response of *E. fetida* may be triggered by the sensory-based reaction, not by the detrimental effect of pollutant uptake.

From our study results, the closed soil microcosm could avoid the influence of the volatilization of volatile compounds on the experimental results, so that the results were more stable and reproducible. Furthermore, the closed soil microcosm required few consumables and simple equipment, so it was more suitable for evaluation of actual contaminated sites. Therefore, the results of this study showed that the closed soil microcosm may be the most suitable for ecotoxicological risk assessment of volatile compounds.

### 3.4. Ecotoxicological Risk Assessment of Three PAs

Based on the 48 h-LC_50_ of the three PAs, the PNEC values of *E. fetida* for the toluene, xylene, and trichloroethylene were calculated as 0.96, 0.22, and 4.52 mg/kg, respectively. According to the investigated reports, the MEC values of toluene and xylene were 0.21~55.20 mg/kg and 0.31~14.80 mg/kg, respectively [45,46]. The RQ values of toluene and xylene were 0.22~57.50 and 1.40~67.30, respectively. Therefore, toluene showed a medium to high ecotoxicological risk to *E. fetida.* The xylene showed high ecotoxicological risk to *E. fetida*. Due to the lack of MEC value of trichloroethylene, the ecotoxicological risk assessment for trichloroethylene could not be carried out.

The present study provided toxicity effect data on the individual level of *E. fetida* to PAs and further evaluated their ecotoxicological risk. The medium to high ecotoxicological risk to *E. fetida* indicated that further studies should be carried out to investigate other toxicity effects, i.e., the biomarkers at a biochemical or biomolecular level and the eco-physiologically differences between different earthworm species.

## 4. Conclusions

(1) The 24 h-LC_50_ of toluene, xylene and trichloroethylene on *E. fetida* were 300.23, 1190.45, and 5332.36 mg/kg, respectively, and the 48 h-LC_50_ of toluene, xylene, and trichloroethylene were 221.62, 962.89, and 4522.41 mg/kg, respectively. The decreasing order of toxicity at the 24 h and 48 h exposure were both: xylene > toluene > trichloroethylene. The ternary mixture exhibited a significant synergistic effect response on *E. fetida*.
(2) The three volatile PAs and their ternary mixture significantly inhibited the growth of *E. fetida*. The significant inhibition concentration and mixing rates were 900 mg/kg, 200 mg/kg, 4000 mg/kg and 25% at 12 h. The AT values of *E. fetida* for the toluene, xylene, and trichloroethylene and their ternary mixture treatments were 1100 mg/kg, 250 mg/kg, 5000 mg/kg and 25%.
(3) By comparing the PNEC values of *E. fetida* for the three volatile PAs and the investigated concentrations in the environment, toluene exhibited a medium to high ecotoxicological risk, and xylene exhibited a high ecotoxicological risk to the ecosystem.

## Figures and Tables

**Table 1:** Chemical information of volatile PAs used in this study.

Chemical	CAS Number	Purity (%)	Water Solubility (mg/L, 25 °C)	Log K_ow_ ^a^
Toluene	108-88-3	99.5	515	2.69
Xylene	1330-20-7	99.0	160	3.12~3.20
Trichloroethylene	79-01-6	99.0	1100	2.42

^a^ Octanol-water partition coefficient.

**Table 2:** The acute toxicity of toluene, xylene, and trichloroethylene to *E. fetida*.

Chemical	Exposure Time (h)	LC_50_ (95% CI ^a^)/(mg/kg)	Regression Equation	R^2^
Toluene	24	1190.45 (1134.75~1283.74)	Y = −37.81 + 12.3X	0.992
	48	962.89 (933.45~991.14)	Y = −46.45 + 15.56X	0.989
Xylene	24	300.23 (284.59~317.15)	Y = −20.45 + 8.26X	0.960
	48	221.62 (202.35~236.66)	Y = −19.47 + 8.30X	0.960
Trichloroethylene	24	5332.36 (4948.86~5679.19)	Y = −26.13 + 7.01X	0.936
	48	4522.41 (4124.26~4833.91)	Y = −29.92 + 8.18X	0.978

Y = Mortality, X = Logarithm of Volatile PAs concentration, R^2^ = Regression equation correlation coefficient, ^a^ Confidence interval.

**Table 3:** The mixed toxicity of toluene, xylene, and trichloroethylene to *E. fetida*.

Exposure Time (h)	LC_50_ (95% CI ^d^)/(mg/kg)			Mixing Rate (95% CI)/(%)	AI (95% CI)	Interaction Type
	**A_m_^a^**	**B_m_^b^**	**C_m_^c^**			
24	270.52 (254.35~290.79)	62.26 (58.54~66.93)	1270.57 (1194.60~1365.77)	28.10 (26.42~30.20)	0.49 (0.38~0.58)	Synergism
48	199.41 (166.53~226.66)	45.90 (38.33~52.17)	936.60 (782.15~1064.58)	20.71 (17.30~23.54)	0.61 (0.42~0.93)	Synergism

^a^ The LC_50_ value in mixture for toluene, ^b^ The LC_50_ value in mixture for xylene, ^c^ The LC_50_ value in mixture for trichloroethylene, ^d^ Confidence interval.

## Data Availability

Data can be obtained by contacting the corresponding author.
